# P-538. People with HIV in the US switching to cabotegravir + rilpivirine long-acting (CAB+RPV LA) from oral antiretroviral therapy (ART) have numerous real-world benefits, including high adherence and treatment satisfaction

**DOI:** 10.1093/ofid/ofae631.737

**Published:** 2025-01-29

**Authors:** Andrew P Brogan, Tim Holbrook, Fritha Hennessy, Will Ambler, Oliver-Thomas Carter, Cindy Garris

**Affiliations:** ViiV Healthcare, San Diego, California; Adelphi Real World, Bollington, United Kingdom, Bollington, England, United Kingdom; Adelphi Real World, Bollington, United Kingdom, Bollington, England, United Kingdom; Adelphi Real World, Bollington, England, United Kingdom; Adelphi Real World, Bollington, England, United Kingdom; ViiV Healthcare, San Diego, California

## Abstract

**Background:**

CAB+RPV LA is a complete HIV regimen which offers less frequent (monthly or every 2 months) dosing than daily oral ART. This real-world study provides additional utilization data of CAB+RPV LA and characterizes the experiences of people with HIV (PWH) receiving CAB+RPV LA in the US.

**Figure:**
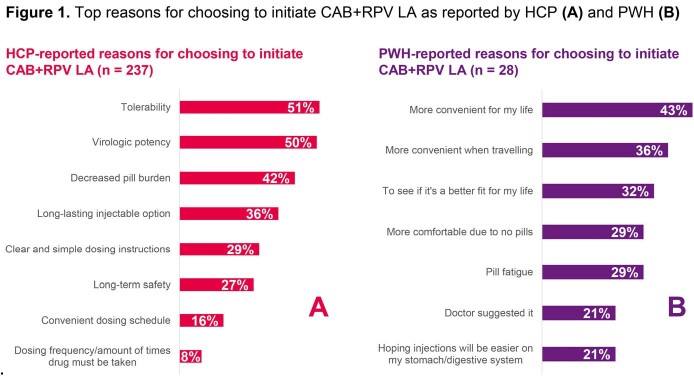

**Methods:**

Data were obtained from the Adelphi HIV Disease Specific Programme™, a real-world, cross-sectional survey containing retrospective longitudinal data of healthcare professionals (HCP) and their PWH on CAB+RPV LA and over two periods (Jul 2021-Mar 2022 and Sept 2023-Feb 2024). HCPs reported demographics, clinical characteristics, and adherence for PWH on CAB+RPV LA aged ≥18 years. PWH surveys included treatment satisfaction, treatment preference and health-related quality of life (HRQoL) (HIV-specific PozQoL [score range 13-65], and EQ-5D-5L-US [score range 0-1]). Data were analyzed descriptively.

**Figure:**
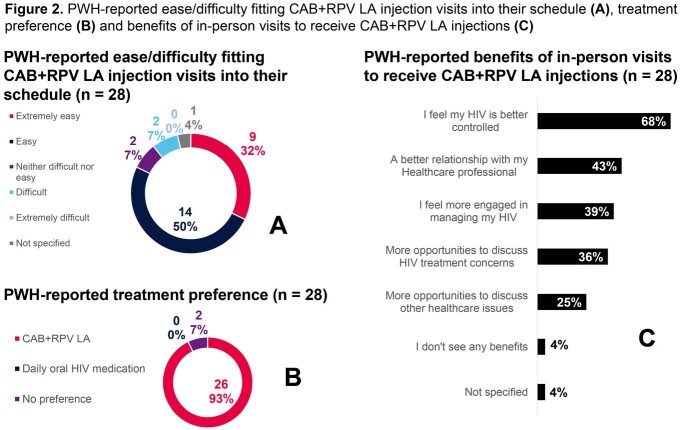

**Results:**

HCPs (n=77) reported data for 237 PWH on CAB+RPV LA treatment for a median [IQR] of 1.0 [0.5, 1.7] years (n=202) at time of data collection, with 65% receiving CAB+RPV LA every 2 months (n=205). Mean age was 42 years, 20% were cisgender female, 43% were non-white (n=205) (Table 1). Top HCP-reported reasons for initiating CAB+RPV LA were tolerability (51%) and virologic potency (50%) (Figure 1). Top PWH-reported reasons for initiating CAB+RPV LA were “more convenient for my life” (43%) and “more convenient when travelling” (36%) (Figure 1). High levels of HCP-reported adherence were seen: 92% (n=205) of PWH received injections either early or within ± 7-day dosing window despite only 64% (n=103) of PWH reported to be completely adherent to prior oral ART. HCP-reported treatment satisfaction was high (93% satisfied/very satisfied). Of PWH who completed a survey, 95% (38/40) were satisfied/very satisfied with CAB+RPV LA. PWH had high HRQoL (PozQoL summary score 49.0 [SD 8.3; n=38] and EQ-5D-5L-US mean 0.88 [SD 0.14], n=39). PWH (82%) reported that fitting injection visits into their schedule was easy/extremely easy (Figure 2). CAB+RPV LA was preferred by 93%, and PWH reported numerous benefits of in-person visits to receive CAB+RPV LA (Figure 2).

**Figure:**
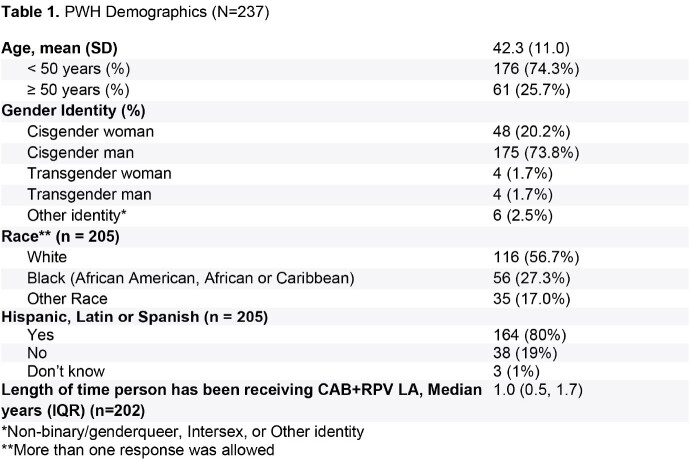

**Conclusion:**

PWH receiving CAB+RPV LA are adherent, highly satisfied, have high HRQoL, prefer LA regimens and reported benefits of in-person visits.

**Disclosures:**

**Andrew P. Brogan, PhD**, GSK: Stocks/Bonds (Public Company)|ViiV Healthcare: Employee **Tim Holbrook, BSc**, Adelphi Real World: Employee|ViiV Healthcare: The Adelphi Real World Disease Specific Programme is wholly owned by Adelphi Real World; ViiV healthcare is one subscriber and paid for the analysis **Fritha Hennessy, PhD**, Adelphi Real World: Employee|ViiV Healthcare: The Adelphi Real World Disease Specific Programme is wholly owned by Adelphi Real World; ViiV healthcare is one subscriber and paid for the analysis **Will Ambler, PhD**, Adelphi Real World: Employee|ViiV Healthcare: The Adelphi Real World Disease Specific Programme is wholly owned by Adelphi Real World; ViiV healthcare is one subscriber and paid for the analysis **Oliver-Thomas Carter, BSc**, Adelphi Real World: Employee|ViiV Healthcare: The Adelphi Real World Disease Specific Programme is wholly owned by Adelphi Real World; ViiV healthcare is one subscriber and paid for the analysis **Cindy Garris, MS**, GSK: Stocks/Bonds (Public Company)|ViiV Healthcare: Employee

